# Oncogenic driver mutations predict outcome in a cohort of head and neck squamous cell carcinoma (HNSCC) patients within a clinical trial

**DOI:** 10.1038/s41598-020-72927-2

**Published:** 2020-10-06

**Authors:** Javier Fernández-Mateos, Jéssica Pérez-García, Raquel Seijas-Tamayo, Ricard Mesía, Jordi Rubió-Casadevall, Carlos García-Girón, Lara Iglesias, Alberto Carral Maseda, Juan Carlos Adansa Klain, Miren Taberna, Silvia Vazquez, María Asunción Gómez, Edel del Barco, Alberto Ocana, Rogelio González-Sarmiento, Juan Jesús Cruz-Hernández

**Affiliations:** 1 Medical Oncology Service, University Hospital of Salamanca-IBSAL, 37007 Salamanca, Spain; 2 Biomedical Research Institute of Salamanca (IBSAL), SACYL-University of Salamanca-CSIC, 37007 Salamanca, Spain; 3 Molecular Medicine Unit-IBSAL, Department of Medicine, University of Salamanca, 37007 Salamanca, Spain; 4 Institute of Molecular and Cellular Biology of Cancer (IBMCC), University of Salamanca-CSIC, 37007 Salamanca, Spain; 5 Medical Oncology Department, Institut Català d’Oncologia, L’Hospitalet de Llobregat, Universitat de Barcelona, IDIBELL, 08908 Barcelona, Spain; 6 Medical Oncology Service, Institut Català d’Oncologia, 17007 Gerona, Spain; 7 Medical Oncology Service, Hospital Universitario de Burgos, 09006 Burgos, Spain; 8 Medical Oncology Service, Hospital Universitario 12 de Octubre, 28041 Madrid, Spain; 9 Medical Oncology Service, Hospital Universitario Lucus Augusti, 27003 Lugo, Spain; 10 Pathologist Service, University Hospital of Salamanca, 37007 Salamanca, Spain; 11 Hospital Clínico San Carlos, IdISSC, CIBERONC, 28040 Madrid, Spain; 12 Centro Regional de Investigaciones Biomédicas, Universidad de Castilla La Mancha, 13071 Albacete, Spain

**Keywords:** Cancer genetics, Genetics, Genomics, Personalized medicine, Cancer, Head and neck cancer

## Abstract

234 diagnostic formalin-fixed paraffin-embedded (FFPE) blocks from homogeneously treated patients with locally advanced head and neck squamous cell carcinoma (HNSCC) within a multicentre phase III clinical trial were characterised. The mutational spectrum was examined by next generation sequencing in the 26 most frequent oncogenic drivers in cancer and correlated with treatment response and survival. Human papillomavirus (HPV) status was measured by p16INK4a immunohistochemistry in oropharyngeal tumours. Clinicopathological features and response to treatment were measured and compared with the sequencing results. The results indicated *TP53* as the most mutated gene in locally advanced HNSCC. HPV-positive oropharyngeal tumours were less mutated than HPV-negative tumours in *TP53* (p < 0.01). Mutational and HPV status influences patient survival, being mutated or HPV-negative tumours associated with poor overall survival (p < 0.05). No association was found between mutations and clinicopathological features. This study confirmed and expanded previously published genomic characterization data in HNSCC. Survival analysis showed that non-mutated HNSCC tumours associated with better prognosis and lack of mutations can be identified as an important biomarker in HNSCC. Frequent alterations in PI3K pathway in HPV-positive HNSCC could define a promising pathway for pharmacological intervention in this group of tumours.

## Introduction

Head and neck squamous cell carcinoma (HNSCC) is the sixth most common neoplasia in the developed world^1^. It constitutes a heterogeneous disease of tumours of the upper aerodigestive tract with different pathogenic origins and clinical prognosis. Tobacco smoking and alcohol consumption are still the most classical risk factors^2^ followed by viral infection^3,4^.

Most HNSCC are diagnosed as locally advanced disease (stage III or IV) and therefore multidisciplinary treatment strategies include surgery, radiotherapy (RT), chemotherapy (CT) and targeted therapy. However, treatment with chemoradiotherapy (CTRT) has become the standard of care after the publication of a large pool analysis^5^. With the aim of improving the clinical benefit, the addition of cetuximab, an IgG1 chimeric monoclonal antibody against epidermal growth factor receptor (EGFR), concomitant with RT was explored, resulting in longer progression-free survival (PFS) and overall survival (OS) compared to RT alone, although a direct comparison with CTRT has not been evaluated yet^6^.

The role of induction chemotherapy has remained a subject of controversy. The combination of docetaxel-cisplatin and 5-fluorouracil (TPF) has emerged as the most active regimen in locally advanced disease, showing better results than PF, although it did not show a convincing survival benefit in induction regimens compared with historical data of treatment with concomitant chemoradiotherapy alone^7–9^. Induction chemotherapy to improve organ preservation and survival may be an alternative to CTRT. The addition of cetuximab to radiation therapy in patients with laryngeal cancer stage III and IVA that respond to TPF could improve functional laryngeal preservation^10^, although randomized phase III trials did not find that induction chemotherapy provided benefit in time-to-treatment failure or OS^11–14^. On the other hand, a randomized phase II–III study suggested that adding TPF induction chemotherapy to CTRT resulted in a higher rate of radiological complete response compared with concurrent CTRT alone, improving PFS and OS by induction TPF^15^. The fact that patient populations in these trials were very heterogeneous, questions induction chemotherapy’s benefit thus, subgroups that will have a benefit from it need to be identified.

Next-generation sequencing (NGS) has helped to identify genetic alterations that could be used as a molecular vulnerability for therapeutic discovery and target optimization. In addition, they could have a prognosis utility as biomarkers of response in different tumour types including head and neck squamous cell carcinomas^16,17^. For instance, the analysis of *The Cancer Genome Atlas* (TCGA) described the molecular landscape of HPV-positive and HPV-negative HNSCC as having molecular alterations not reported before^18^. Since the first description of the recurrently mutated genes in HNSCC^19^, additional studies have included other genes such as *TP53, NOTCH1, PIK3CA, CDKN2A, CCDN1, HRAS, FAT1, FBXW7* and *FGFR3,* among others^20,21^. For this reason, targeted sequencing has become a flexible tool to study those genes previously reported as mutated in HNSCC^21^.

To contribute to the understanding of how somatic mutations influence the outcome of HNSCC treatment, we have studied a panel of 26 genes (Table S1) by next-generation sequencing in a homogenously treated locally advanced HNSCC Spanish cohort. In this study we report some mutations linked with detrimental outcome and their presence in relation to HPV presence.

## Results

### Cohort characteristics

234 FFPE blocks with diagnostic biopsies from HNSCC patients within a multicentre phase III clinical trial were incorporated in this study (Fig. S1). Clinical demographic factors such as age, gender, disease site and tumour stage are consistent between the whole cohort within the clinical trial and the subsequent random selection due to FFPE block availability in this study. Overall, most were from men (89.7%), with pharyngeal carcinoma (65.4%) and diagnosed in tumour stage IV-A (71.4%) with an average of 57 years old (Table 1). Clinicopathologic features by locations are shown in Table 1.

**Table 1:** Clinicopathologic characteristic of the 234 HNSCC patients included in the study: overall and by subtypes.

Overall		Subtypes			
Variable	N (%)	Oropharynx	Hypopharynx	Larynx	Oral cavity
		N (%)	N (%)	N (%)	N (%)
	234 (100)	90 (38.5)	63 (26.9)	41 (17.5)	40(17.1)
**Age (years ± SD)**	57.42 ± 6.9	59.46 ± 6.7	56.61 ± 8.1	58.34 ± 5.7	57.55 ± 6.9
**Sex**					
Man	209 (89.7)	76 (36.4)	59 (28.2)	40 (19.1)	34 (16.3)
Woman	24 (10.3)	14 (58.3)	3 (12.5)	1 (4.2)	6 (25.0)
Unk	1	0	1	0	0
**TNM**					
III	20 (8.7)	11 (55.0)	1 (5.0)	4 (20.0)	4 (20.0)
IVA	165 (71.4)	64 (38.8)	43 (26.1)	29 (17.6)	29 (17.6)
IVB	46 (19.9)	15 (32.6)	17 (37.0)	8 (17.4)	6 (13.0)
Unk	3	0	2	0	1
**Grade**					
Well differentiated	32 (15.1)	13 (40.6)	8 (25.0)	3 (9.4)	8 (25.0)
Moderately differentiated	102 (48.1)	41 (40.2)	15 (14.7)	27 (26.5)	19 (18.6)
Poorly differentiated	78 (36.8)	28 (35.9)	32 (41.0)	9 (11.5)	9 (11.5)
Unk	22	8	8	2	4
**Histology**					
Keratinizing	94 (48.0)	40 (42.6)	19 (20.2)	13 (13.8)	22 (23.4)
Non-keratinizing	100 (51.0)	36 (36.0)	31 (31.0)	25 (25.0)	8 (8.0)
Undifferentiated	2 (1.0)	1 (50.0)	0 (0)	0 (0)	1 (50.0)
Unk	38	13	13	3	9
**HPV**					
Positive	13 (6.7)	13 (100.0)	0 (0)	0 (0)	0 (0)
Negative	182 (93.3)	63 (34.6)	50 (27.5)	38 (20.9)	31 (17.0)
Unk	39	14	13	3	9

TNM classification system stands for tumour, node and metastasis.

*S*D standard deviation, *Unk* unknown.

Considering only oropharyngeal tumours (see “Methods” section), 13 samples (17.1%) were HPV-positive based on p16 immunohistochemistry (IHC). According to its grade, HPV-positive samples were statistically associated with poorly differentiated (p = 0.016) and *TP53* wild-type (p = 0.009) tumours (Table 2).

**Table 2:** HPV association in oropharyngeal tumours with clinicopathological characteristics.

Variable	HPV-negative	HPV-positive	p-value
	N (%)	N (%)	
	63 (82.9)	13 (17.1)	
**Age (years ± SD)**	57.08 (7.11)	58.46 (6.40)	0.562*^1^
**Sex**			
Man	55 (84.6)	10 (15.4)	0.388*^2^
Woman	8 (72.7)	3 (27.3)	
**TNM**			
III	5 (71.4)	2 (28.6)	0.166*^3^
IVA	45 (80.4)	11 (19.6)	
IVB	13 (100.0)	0 (0.0)	
**Grade**			
Well differentiated	10 (83.3)	2 (16.7)	0.016*^3^
Moderately differentiated	36 (94.7)	2 (5.3)	
Poorly differentiated	16 (64.0)	9 (36.0)	
**Histology**			
Keratinizing	36 (90.0)	4 (10.0)	0.177*^3^
Non-keratinizing	26 (74.3)	9 (25.7)	
Undifferentiated	1 (100.0)	0 (0.0)	
**Mutational status**			
Non-mutated	15 (78.9)	4 (21.1)	0.726*^2^
Mutated	48 (84.2)	9 (15.8)	
**TP53 mutated**			
Non-mutated	18 (66.7)	9 (33.3)	0.009*^2^
Mutated	45 (91.8)	4 (8.2)	
**PIK3CA mutated**			
Non-mutated	57 (85.1)	10 (14.9)	0.178*^2^
Mutated	6 (66.7)	3 (33.3)	

TNM classification system stands for tumour, node and metastasis.

HPV status based on p16 + IHC could only be measured in 76 (84.4%) out of the 90 oropharyngeal tumour samples.

*SD* standard deviation, *Unk* unknown.

*^1^Mann-Whitney U test.

*^2^Fisher’s exact test.

*^3^Chi-square test.

**Figure 1:**
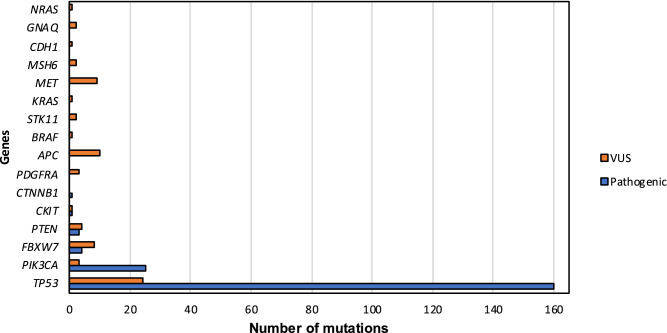
Number of mutations found in the sequencing of 234 HNSCC by TruSight Tumor 26 panel. Blue bars represent pathogenic mutations while orange bars show variants of uncertain significance (VUS).

Targeted panel sequencing in HNSCC FFPE blocks identified 162 samples (69.23%) with previously described pathogenic mutations whereas 46 (19.66%) did not carry any mutation and 26 (11.11%) showed variants of uncertain clinical significance (VUS). 194 pathogenic mutations and 72 VUS were found in the sequencing of the 234 FFPE blocks. All samples were sequenced > 5000 × (7074 ± 10,516). Globally, the most mutated gene was *TP53* (61.1%) followed by *PIK3CA* (10.3%), *FBXW7* (1.7%), *PTEN* (1.3%) and *CKIT* and *CTNNB1* (both with 0.43%) (Fig. 1). 144 out of 162 (88.89%) mutated tumours had *TP53* mutations either alone or with others. Most of the pathogenic variants were missense (55.67%), followed by stop-gained (18.04%), frameshift (14.95%), splice-donor (8.76%) and in-frame deletions (2.58%) (Table S2).

### Association of mutations with clinical variables

General comparison of the mutational status and tumour characteristics such as location, grade and histology, did not show any significant difference (p > 0.05) (Table 3). However, considering variants of uncertain significance, women were associated with a lower percentage of mutation than men in our cohort (p = 0.002) (Table 3).

**Table 3:** Association between mutational status and clinicopathological characteristics.

Variable	Normal	Pathogenic	VUS*	p-value^a^	p-value^b^
	N (%)	N (%)	N (%)	All	Normal vs mutant
	46 (19.66)	162 (69.23)	26 (11.11)		
**Age (years ± SD)**	57.02 (7.65)	57.68 (6.95)	56.44 (6.50)	0.683*^1^	0.941*^1^
**Sex**					
Man	39 (18.7)	152 (72.7)	18 (8.6)	0.002*^2^	0.290*^2^
Woman	7 (29.2)	10 (41.7)	7 (29.2)		
Unk	0	0	1		
**TNM**					
III	5 (25.0)	13 (65.0)	2 (10.0)	0.777*^2^	0.781*^2^
IVA	32 (19.4)	113 (68.5)	20 (12.1)		
IVB	8 (17.4)	35 (76.1)	3 (6.5)		
Unk	1	1	1		
**Location**					
Oropharynx	16 (17.8)	66 (73.3)	8 (8.9)	0.644*^2^	0.582*^2^
Hypopharynx	14 (22.2)	43 (68.3)	6 (9.5)		
Larynx	8 (19.5)	29 (70.7)	4 (9.8)		
Oral cavity	8 (20.0)	24 (60.0)	8 (20.0)		
**Grade**					
Well differentiated	7 (21.9)	24 (75.0)	1 (3.1)	0.578*^2^	0.588*^2^
Moderately differentiated	17 (16.7)	73 (71.6)	12 (11.7)		
Poorly differentiated	17 (21.8)	52 (66.7)	9 (11.5)		
Unk	5	13	4		
**Histology**					
Keratinizing	14 (14.9)	72 (76.6)	8 (8.5)	0.346*^2^	0.652*^2^
Non-keratinizing	21 (21.0)	67 (67.0)	12 (12.0)		
Undifferentiated	1(50.0)	1(50.0)	0 (0.0)		
Unk	10	22	6		

TNM classification system stands for tumour, node and metastasis.

*SD* standard deviation, *Unk* unknown.

^a^Initially, statistical analysis was done comparing normal, pathogenic mutation and variants of uncertain clinical significance (VUS), p-value.

^b^To avoid bias with VUS, a direct comparison only between normal and pathogenic mutation was done, p-value.

*Variant of uncertain clinical significance.

*^1^Kruskal–Wallis H test.

*^2^Fisher’s exact test.

*^3^Chi-square test.

### Mutational profile and HPV presence in oropharyngeal tumours

**Figure 2:**
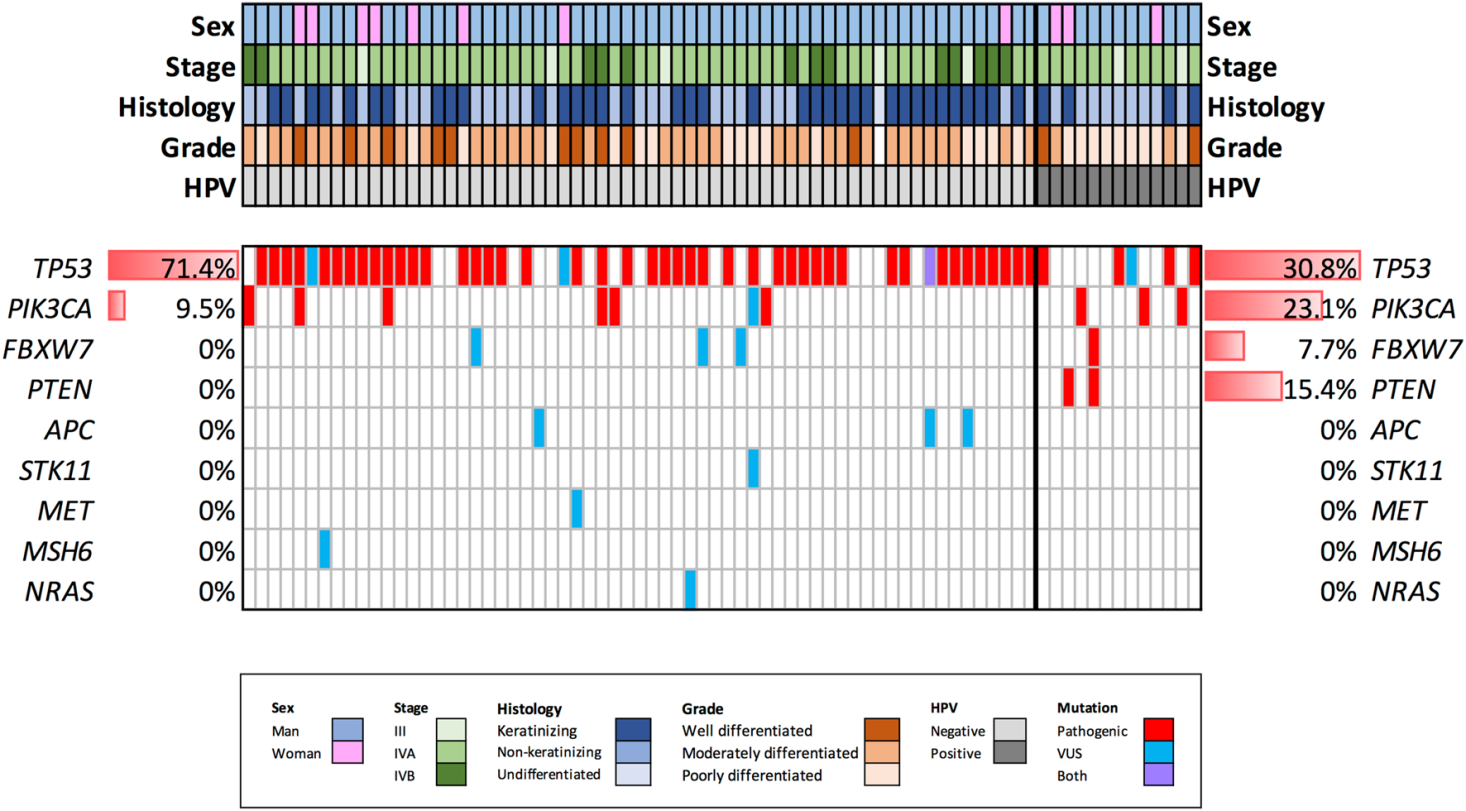
Mutational landscape plot divided into HPV-negative (left) and HPV-positive (right) oropharyngeal tumours. Legend represents different colours according to its clinical features. Percentage of pathogenic mutations in each gene is indicated at the edges by their HPV profile.

HPV mutational profile in oropharyngeal tumours is shown in Fig. 2. HPV-positive samples presented slightly more pathogenic mutations than HPV-negative (76.2% versus 69.2%, p = 0.762) (Table 2). Despite the fact that *TP53* was the most frequently mutated gene in both groups, these mutations were more recurrent in HPV-negative tumours (71.4% in HPV-negative and 30.8% in HPV-positive), difference statistically significant (p = 0.009). Conversely, the second most mutated gene, *PIK3CA,* although more represented in HPV-positive tumours (9.5% in HPV-negative versus 23.1% in HPV-positive), did not show any statistically significant difference (p = 0.178). While HPV-negative tumours did not present pathogenic mutations in other genes, *PTEN* was the third most commonly mutated in HPV-positive tumours (15.4%), followed by *FBXW7* (7.7%).

### Mutational status and response to treatment

After induction chemotherapy, 188 (80.34%) patients were similarly randomized: 95 (50.53%) to conventional treatment and 93 (49.47%) to the experimental arm. Preliminary data indicated that the two regimens showed similar survival, response rates, toxicity and locoregional control^22^. For that reason, both arms were evaluated within the same group as final response (or response after randomization). Evaluation of the two time-point responses according to the mutational profile did not show any statistical difference (Table 4). There was, however, a tendency between mutated tumours and complete response at the end of the treatment taking VUS into consideration, p = 0.096 (Table S3).

**Table 4:** Association between mutational status and response after induction and randomization (final response), without VUS.

Variable	Normal	Pathogenic	OR	95% CI	p-value
	N (%)	N (%)			
**Response after induction**	37 (21.5)	135 (78.5)			
Complete	5 (13.5)	19 (14.1)	0.954	0.330–2.753	0.931
Partial/stabilization	32 (86.5)	116 (85.9)			
**Final response**	21 (17.9)	96 (82.1)			
Complete	13 (61.9)	61 (63.5)	0.932	0.352–2.469	0.888
Partial/stabilization	8 (38.1)	35 (36.5)			

p-value significant if p < 0.05 and size effect indicated by the odd ratio (OR) with 95% confidence interval (CI). 172 (73.5%) and 117 (50.0%) patients were evaluable after induction chemotherapy or randomization respectively.

Considering only HPV profile in oropharyngeal tumours, there were no differences between HPV-positive and HPV-negative individuals either after induction chemotherapy (p = 0.396) or randomization (p = 0.914) (Table 5).

**Table 5:** Association between HPV status and response after induction and randomization (final response).

Variable	HPV-negative	HPV-positive	OR	95% CI	p-value
	N (%)	N (%)			
**Response after induction**	52 (80.0)	13 (20.0)			
Complete	7 (70.0)	3 (30.0)	0.519	0.114–2.362	0.396
Partial/stabilization	45 (81.8)	10 (18.2)			
**Final response**	33 (78.6)	9 (21.4)			
Complete	19 (79.2)	5 (20.8)	1.086	0.246–4.793	0.914
Partial/stabilization	14 (77.8)	4 (22.2)			

p-value significant if p < 0.05 and size effect indicated by the odd ratio (OR) with 95% confidence interval (CI). From initial 76 oropharyngeal tumours with HPV determination, 65 (85.5%) of them were evaluable for response after induction and 42 (55.3%) for final response.

Finally, an exploratory analysis was performed using the two most mutated genes in the study: *TP53* and *PIK3CA* (Table S4). Analysing patients with mutations in those genes alone or within other genes and the clinical response, indicated that none of the *TP53* subgroups were associated in any of the clinical trial treatment timepoints (p > 0.05). By contrast, considering only *PIK3CA* mutations, a statistically positive association was found in the complete response group after induction chemotherapy (p = 0.024). However, this finding was not corroborated in final response group (p = 0.235) (Table S4) what could suggest that this could be a false positive result taken into consideration multiple testing and sample size bias.

Poeta’s^23^ and Neskey’s^24^ classification in patients harbouring *TP53* mutations in relation with clinical response before and after randomization did not show any statistically significant association (p > 0.05, Table S5).

### HPV, mutational status and clinical outcome

**Figure 3:**
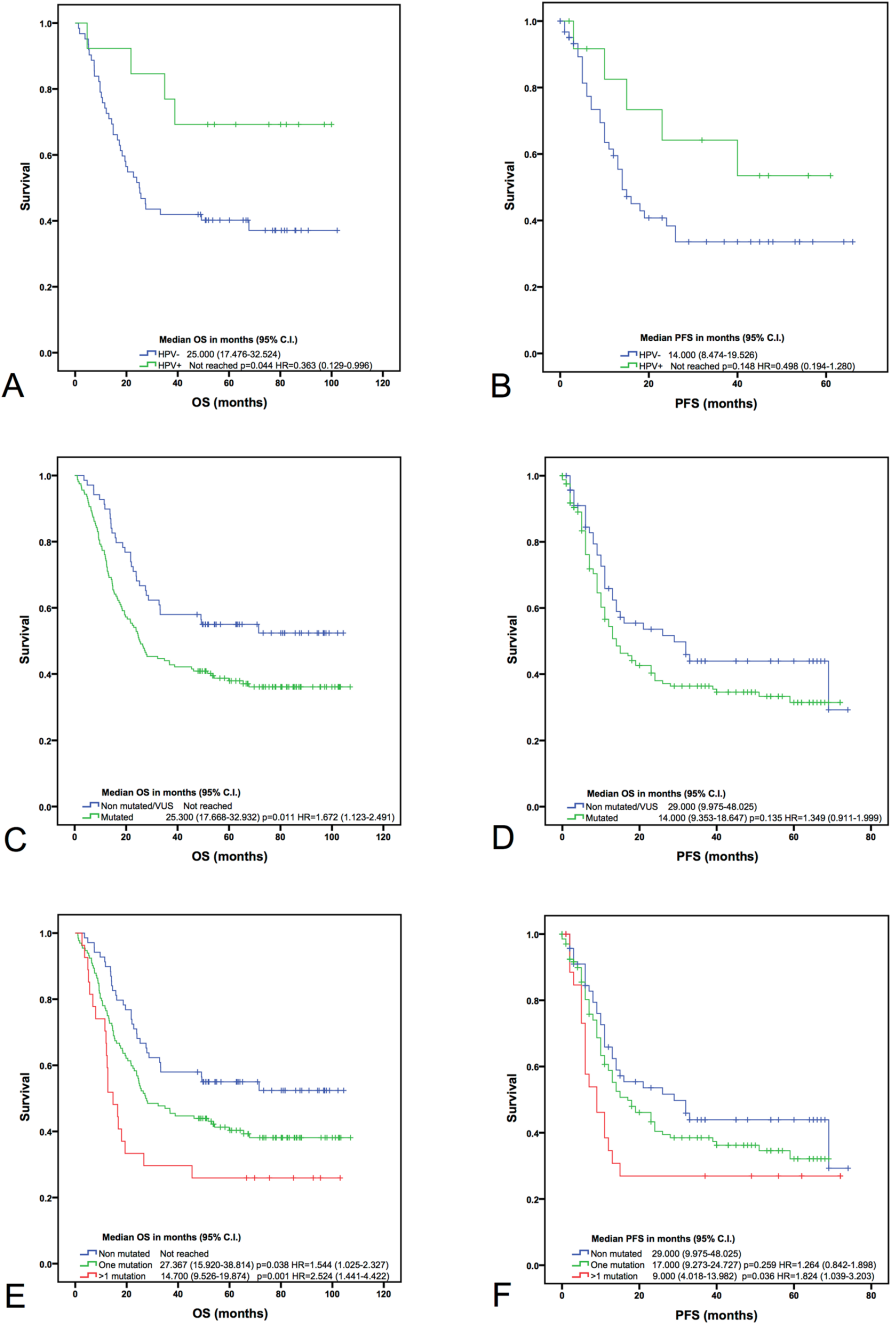
Kaplan–Meier survival curves. HPV status in oropharyngeal tumours and overall survival (OS) (**A**) and progression free survival (PFS) (**B**), mutational status in all the samples and OS (**C**) and PFS (**D**), number of mutations in all the samples and their OS (**E**) and PFS (**F**). Median with 95% confidence interval (CI), log rank test p-values and hazard ratios (HR) with 95% CI, are shown in each plot.

HPV-positive oropharyngeal tumours showed higher OS compared with HPV-negative (p = 0.044). This tendency was also shown in PFS, however, without statistically significant results (p = 0.148, HR = 0.498 (0.194–1.280)) (Fig. 3A,B).

Moreover, OS was correlated with the mutational status. Patients without mutations in the selected genes had a better OS than patients with mutated tumours (p = 0.011, HR = 1.672 (1.123–2.491)) (Fig. 3C). This difference was also observed in PFS without statistically significant results (p = 0.135, HR = 1.349 (0.911–1.999)) (Fig. 3D).

A correlation with the number of mutations also showed that tumours with one mutation had lower OS (p = 0.038, HR = 1.544 (1.025–2.327)) than non-mutated patients, with the exception of PFS (p = 0.259, HR = 1.264 (0.842–1.898)) (Fig. 3E,F). Equally, tumours with more than one mutation showed lower OS (p = 0.001, HR = 2.524 (1.441–4.422)) and PFS (p = 0.036, HR = 1.824 (1.039–3.203)) than non-mutated samples. Conversely, the differences between tumours with one or more mutations were not statistically significant (p > 0.05).

Finally, we compared *TP53* mutations based on Poeta’s^23^ and Neskey’s^24^ stratification models with OS and PFS (Fig. S2). No association was observed between low-risk/high risk mutations or non-disruptive/disruptive mutations and survival in these patients (Fig. S2).

## Discussion

As most of the head and neck cancers are diagnosed at a locally advanced stage the identification of biomarkers of response is a main goal to optimize treatment and reduce side effects. In recent years, induction chemotherapy has been shown to produce a benefit in organ preservation without a clear improvement in survival. In addition, this approach led to a high toxicity, particularly when concurrent radiotherapy was given with high doses of cisplatin. At present, very few predictive biomarkers of response have been described. For this reason, we proposed a study of the mutational status in 26 of the most common altered genes in cancer with next-generation sequencing in a homogeneously treated representative Spanish cohort of HNSCC from the phase III clinical trial TTCC-2007-01^22^.

The epidemiology characteristics of the HNSCC patients included in our study were similar to other series reported from the same region: the ratio between sexes is 9:1 in detriment of men, and most of the patients were diagnosed at stage IV^25^. p16 IHC, a surrogate of HPV infection in oropharyngeal tumours, showed that HPV was present in 17.1% of samples, a lower percentage than previously reported in Europe^26^ but with similar location to other Southern European countries in oropharynx^27^.

Globally, the most mutated gene in our series was *TP53* (61.1%). We observed a statistically significant lower percentage of mutated *TP53* in HPV-positive oropharyngeal tumours (71.4%) than in HPV-negative (30.8%) as has been previously reported in HNSCC^28,29^. These results could be explained if *TP53* sequestration by the viral oncoprotein E6 prevents gaining mutations in this gene under selective pressure of^30,31^. Comparing to other series, there was a higher percentage of *TP53* mutations in HPV-positive tumours^29^. This fact could be explained by the coexistence of viral infection and other aetiological factors such as tobacco smoking and alcohol consumption during tumourigenesis^32^; these data were not collected in this study. TCGA data described 85% of *TP53* mutation in HPV-negative tumours and only 3% in HPV-positive ones^18^. However, the sample population was very different with a high predominance of oral cavity tumours (62%) and mainly heavy smokers.

*PI3K/AKT/mTOR* has been reported as the most mutated pathway in HNSCC (13% to 56%), regardless of the HPV status^18^. *PIK3CA* gene, that encodes the catalytic subunit of the family, has been reported with an average mutational rate of 10.53% in HNSCC^33^, similar to the 10.25% found in this cohort, and with a higher frequency in laryngeal tumours^34^. Mutations in this gene have also been related to HPV-positive tumours^4^. Our results corroborate this fact, being *PIK3CA* more frequently mutated in HPV-positive tumours (23.1% versus 9.5% in HPV-negative oropharyngeal tumours), similar to previously described data^35^. We did not, however, see an increased percentage in laryngeal carcinoma. 73% of the mutations in *PIK3CA* are commonly located in 3 hotspots (E542K, E545K and H1047R/L)^36^, result also found in 76% of *PIK3CA* mutated samples in our study, emphasising the accuracy of using the targeted panel in HNSCC.

Mutations in *FBXW7*: an E3 ubiquitin ligase member of the F-box protein family, have been previously observed in HNSCC^19^. This tumour suppressor gene targets *NOTCH1*, being an important protein in cell proliferation control. Previous studies found *FBXW7* mutated in 5% of HNSCC^37,38^ and a higher percentage of mutations was previously considered as a prevalent event in HPV-positive tumours^39^. Our cohort confirmed these results in *FBXW7* with a similar percentage only found in HPV-positive tumours (7.7%).

*PTEN* was the third most mutated gene in 15.4% of the HPV-positive oropharyngeal tumours while not mutations were found in HPV-negative ones. Contrary to our results, TCGA study showed *PTEN* mutated in 12% of HPV-negative tumours and 6% of HPV-positive^18^. Apart from *PTEN*, there were other genes which mutated at a lower percentage in our series, such as *CKIT* or *CTNNB1* (both mutated at less than 1% and only in non-oropharyngeal HPV-negative tumours)*,* have been reported in HNSCC in varied percentages^30,38,40^. Together with *PIK3CA*, our result enhances the hypothesis of higher prevalence of PI3K pathway activated mutations in HPV-positive tumours^41^.

Overall, excluding *TP53* mutations, recurrent alterations in *PIK3CA, PTEN* and *FBXW7* genes, all belonging to the *PI3K/AKT/mTOR* pathway, could define a potential new target for pharmacological intervention in HNSCC, as it has been suggested in other publications^42^.

In terms of survival, HPV-positive oropharyngeal tumours were associated with better prognosis, showing an increased OS and PFS compared to HPV-negative tumours as it was previously defined^26,43–47^. Secondly, the presence of mutation in the targeted genes was associated with inferior outcome demonstrated by the presence of detrimental OS. These results could be an indirect measure of tumour aggressiveness, as has been reported in other series^43,47^. Moreover, the fact that carriers of tumours with more than one mutation have lower OS than those with non-mutated tumours reinforces this concept.

Lastly, there was a lack of association between mutational status and response after treatment. This can indicate that, excluding genetic-driven druggable targets, HNSCC mutational profile is not related to any clinical response but is a matter of mutational burden as is shown in the survival analyses. Similarly, there was no association between *TP53* mutations stratified by Poeta’s^23^ and Neskey’s models^24^ and response to treatment or survival. These classification systems can serve as an important tool in individualizing and improving treatment for high TP53 mutated tumours, as it was previously identified in a subset of high-risk patients with a decreased response to platinum-based therapies^48^. Nevertheless, these classification models did not have any implication on outcome in our cohort.

Overall, our data strongly support and expand previously published studies exploring the presence and prognosis of mutations in this population. We have characterized the mutational profile of HPV-positive/HPV-negative oropharyngeal HNSCC in a representative cohort of patients. In this context apart from *TP53* mutations, frequent alterations in *PIK3CA, PTEN* and *FBXW7* genes, define possible pathways for pharmacological intervention. Finally, survival analysis showed that mutational status in the tumour could define patient prognosis, and may potentially be used as biomarkers to stratify patients for more intensive treatment. However, larger studies should be performed to confirm these results aiming at stratifying patients to different therapeutic interventions.

## Methods

### Samples

234 FFPE blocks with diagnostic biopsies from HNSCC patients were included in this study. A consort diagram reporting the dropout is shown in Fig. S1. All samples belong to the clinical trial TTCC-2007-01 entitled: “Open label randomized, multi-centre phase III trial of TPF plus concomitant treatment with cisplatin and radiotherapy versus concomitant cetuximab and radiotherapy in locally advanced, unresectable head and neck cancer”, ClinicalTrials.gov identifier: NCT00716391^22^.

### TTCC-2007-01 trial design and data collection

It was a non-inferiority, randomized and controlled study with a parallel assignment intervention model and an endpoint of safety/efficacy, carried out between 2008 and 2013. The follow-up of the clinical trial finished in November 2016. According to protocol, written informed consent was obtained from living subjects and the protocol was approved by the University Hospital of Salamanca and the ethical committees of each hospital in accordance with the 1964 Helsinki declaration and its later amendments.

Eligible patients: histologically or cytologically confirmed, previously untreated unresectable locally advanced (Stage III–IV) tumours (from oral cavity, oropharynx, larynx, hypopharynx), ECOG performance status 0–1. Unresectable disease was determined by Northern California Oncology Group in measurable disease. Treatment: docetaxel, cisplatin, 5-fluorouracil (TPF)-based induction chemotherapy (T 75 mg/m^2^ d1, P 75 mg/m^2^ d1, F 750 mg/m^2^ CI d 1–5 q 21 d + G-CSF & ciprofloxacin, by 3 cycles; then, if objective response achieved, they were randomized to: conventional radiotherapy (RT) up to 70 Gy + P 100 mg/m^2^ d 1–22–43 vs conventional RT up to 70 Gy + cetuximab 400/250 mg/m^2^ weekly until the completion of RT, and they were stratified by primary tumour site. Surgery after RT (neck dissection) was allowed. The primary endpoint was non-inferiority of cetuximab-radiotherapy versus cisplatin-radiotherapy in terms of overall survival. Response rate, loco-regional control and toxicity in both arms were considered secondary objectives. Preliminary data of this trial did not show any difference in terms of survival or response rates, toxicity and loco-regional control as secondary end points in the two regimens^22^.

Clinical data were compiled in a case report form by medical oncologists involved in the clinical trial. All data were treated with the security measures established in compliance with the Protection of Personal Data Organic Law 15/1999, 13th December, and safe-keeping at the University Hospital of Salamanca in its specific server.

### DNA extraction

Percentage of tumour cells was measured in haematoxylin–eosin tissue sections by central pathologist. Between four and ten 10 µm FFPE sections from diagnosis blocks were treated with deparaffinization solution (Qiagen, Hilden, Germany) and DNA extraction was done using QIAamp DNA FFPE Tissue kit (Qiagen, Hilden, Germany).

### DNA quality evaluation and targeted NGS

Following TruSight Tumor 26 Reference Guide (Illumina, San Diego, USA), DNA quality was measured by qPCR. Comparing FFPE-gDNA amplification potential with a reference non-FFPE gDNA (QCT), delta Cq value was used to predict the dilution required for each sample.

TruSight Tumor 26 panel includes a set of 174 amplicons in complete exons of 26 cancer-associated genes (Table S1). This panel was selected due to its exceptional success rate using minimal DNA input even from FFPE samples where genetic material is often degraded. Following steps of hybridization with the oligo pool, removing unbound oligos and extension and ligation with bound oligos, an amplification of the libraries were performed. PCR products were checked on a 4% TBE agarose gel and finally the libraries were cleaned up by AMPure XP magnetic beads (Beckman Coulter, Brea, CA, USA). PCR products were quantified using Qubit Fluorometer (Invitrogen, Carlsbad, CA, USA) and libraries were normalized at 4 nM in a final pool. Sequencing was performed in a NextSeq 500 System (Illumina, San Diego, USA).

Data were transformed in BaseSpace platform and the VCF file format were read in the Variant Studio Software (Illumina, San Diego, USA). Following Illumina recommendations, somatic variants over 5% of frequency, with yields at least 1000 × cumulative coverage between the 2 strands and considered from the software of PASS filter were reported. Those variants of uncertain significance were considered pathogenic if at least two in silico prediction tools (SIFT and PolyPhen) classified them as deleterious/probably damaging^49^, and they were defined as likely pathogenic in the Catalogue Of Somatic Mutations in Cancer (COSMIC; https://cancer.sanger.ac.uk/cosmic) or the National Center for Biotechnology Information (NCBI; https://www.ncbi.nlm.nih.gov/clinvar) databases.

### Assessment of HPV status

In the original study protocol, the assessment of HPV status was carried out by p16 immunohistochemistry (IHC), a surrogate marker for HPV infection^50^ as the gold-standard technique. FFPE sections were deparaffinized and exposed to 10 mM citrate buffer antigen retrieval at 92 °C for 30 min and then they were stained using a p16^INK4a^ mouse monoclonal antibody (Cell Marque, Rocklin, CA, USA). Percentage of p16 staining was measured and only those tumours > 70% nuclear and cytoplasmic p16+ were considered positive. 33 samples were considered HPV-positive following this methodology: 13 oropharyngeal, 4 hypopharyngeal, 2 laryngeal and 9 oral cavity tumours. However, after the publication of the guidelines from the college of American pathologists, p16 IHC is only recommended in oropharyngeal tumours but other locations, where DNA/RNA viral determination should be performed as a confirmatory test^51^. Since there was not more DNA from all the samples after the library preparation, only oropharyngeal tumours with > 70% p16 positive staining were considered HPV-positive.

### Statistical analyses

Statistical analysis compared categorical parameters and mutational status by the Chi-square or Fisher’s exact tests; while in continuous nonparametric variables, the Mann–Whitney U or Kruskal–Wallis H tests were used. p-values were calculated excluding missing values and they were considered statistically significant when p < 0.05. Significant variables were included in the logistic regression analysis and size effects were indicated by odds ratio (OR) with their 95% confidence interval (95% CI). Mutational status was classified as presence or absence of mutations, number of mutations (none, one or more than one) and the status of *TP53* and *PIK3CA* (mutant or wild-type). Response was divided in two groups of treatment: after induction chemotherapy and after chemo/cetuximab plus radiotherapy (final response) due to the similar outcome in both arms^22^. Response was classified in both groups as complete response versus partial response/stabilization. No progressions were shown in the cohort.

Survival analysis was done according to the overall survival (OS) and progression-free survival (PFS) by Kaplan–Meier plots and log-rank test p-values were calculated in all the curves. Median was indicated in those plots in which it was achieved. Hazard-ratio was calculated to measure the risk of the event with its 95% confidence interval (95% CI) by Cox regression. Median follow-up in OS was 32.23 months while in PFS it was 15.31 months.

Due to high prevalence in *TP53* mutations, we applied Poeta’s and Neskey’s classifications stratifying the mutations according to its change and functional effect, allowing a better comprehensive understanding on their relevance in clinical outcome. Following Poeta’s classification^23^, *TP53* mutations were divided in two categories: disruptive and non-disruptive according to their functional effects on the p53 protein. Additionally, according to Neskey’s model^24^, also named as Evolutionary Action score of *TP53*-coding variants (EAp53), missense mutations were stratified into high-risk and low-risk through an *in-silico* scoring (https://mammoth.bcm.tmc.edu/EAp53/). Then, comparative analysis was performed in response to treatment, OS and PFS.

All these tests were conducted using SPSS software version 21.0 (SPSS Inc., Chicago) and GraphPad Prism software version 6.0 (GraphPad Software Inc., California).

## Supplementary information

Supplementary Information 1.

Supplementary Information 2.
