# Supplementary material for: Oncogenic driver mutations predict outcome in a cohort of head and neck squamous cell carcinoma (HNSCC) patients within a clinical trial
Source: Sci Rep. 2020 Oct 6;10:16634. doi: 10.1038/s41598-020-72927-2 (PMC7539152; doi:10.1038/s41598-020-72927-2)
Supplement: Supplementary file 1 — Supplementary Information 1. [file 41598_2020_72927_MOESM1_ESM.docx]

*Supplementary Materials*

Oncogenic driver mutations predict outcome in a cohort of Head and Neck Squamous Cell Carcinoma (HNSCC) patients within a clinical trial

Javier Fernández-Mateos ^1,2,3,4^, Jéssica Pérez-García ^3,4^, Raquel Seijas-Tamayo ^1,2^, Ricard Mesía ^5^, Jordi Rubió-Casadevall ^6^, Carlos García-Girón ^7^, Lara Iglesias ^8^, Alberto Carral Maseda ^9^, Juan Carlos Adansa Klain ^1,2^, Miren Taberna ^5^, Silvia Vazquez ^5^, María Asunción Gómez ^10^, Edel del Barco ^1,2^, Alberto Ocana ^11,12^, Rogelio González-Sarmiento ^2,3,4^*, Juan Jesús Cruz-Hernández ^1,2,3,4^*

^1^ Medical Oncology Service, University Hospital of Salamanca-IBSAL, Salamanca, 37007 Spain

^2^ Biomedical Research Institute of Salamanca (IBSAL), SACYL-University of Salamanca-CSIC, Salamanca, 37007, Spain

^3^ Molecular Medicine Unit- IBSAL, Department of Medicine, University of Salamanca, 37007, Spain

^4^ Institute of Molecular and Cellular Biology of Cancer (IBMCC), University of Salamanca-CSIC, Salamanca, 37007, Spain

^5^ Medical Oncology Department, Universitat de Barcelona, IDIBELL, Institut Català d'Oncologia, L'Hospitalet de Llobregat, Barcelona, 08908, Spain

^6^ Medical Oncology Service, Institut Català d'Oncologia, Girona, 17007, Spain

^7^ Medical Oncology Service, Hospital Universitario de Burgos, Burgos, 09006, Spain

^8^ Medical Oncology Service, Hospital Universitario 12 de Octubre, Madrid, 28041, Spain

^9^ Medical Oncology Service, Hospital Universitario Lucus Augusti, Lugo, 27003, Spain

^10^ Pathologist Service, University Hospital of Salamanca, 37007, Spain

^11^ Hospital Clínico San Carlos, IdISSC, CIBERONC, 28040 Madrid, Spain

^12^ Centro Regional de Investigaciones Biomédicas, Universidad de Castilla La Mancha, 13071 Albacete, Spain

***** Correspondence: gonzalez@usal.es; Tel.: +34 923294553 (R.G.S.)

jjcruz@usal.es; Tel.: +34-923291100 Ext: 55749 (J.J.C.H.)

**Table S1.** Analysed regions in TruSight Tumor 26 panel.

| AKT exon 2 | ALK exon23 | APC exon 15 | BRAF exons 11,15 |
| --- | --- | --- | --- |
| CDH1 exons 8,9,12 | CTNNB1 exon 2 | EGFR exons 18,19,20,21 | ERBB2 exon 20 |
| FBXW7 exons 7,8,9,10,11 | FGFR2 exon 6 | FOXL2 exon 1 | GNAQ exons 4,5,6 |
| GNAS exons 6,8 | KIT exons 7,8,9,13,17,18 | KRAS exons 1,2,3,4 | MAP2K1 exon 2 |
| MET exons 1, 4, 13,15, 16, 17, 18, 20 | MSH6 exon 5 | NRAS exons 1,2,3,4 | PDGFRA exon 11,13,17 |
| PIK3CA exons 1,2,7,9,20 | PTEN exons 1, 2, 3, 4, 5, 6, 7, 9 | SMAD 4 exons 8,9 | SRC exon 10 |
| STK11 exons 1,4,6,8 | | TP53 exons 2,3,4,5,6,7,8,9,10,11 | |

**Table S2.** Excel file list of pathogenic mutations and VUS found in the studied cohort.

**Table S3.** Association between mutational status and response after induction and randomization (final response). Not available response data in 39 samples after induction and 51 samples after randomization.

**Variable Complete Partial/stabilization** **OR 95% CI P N (%) N (%)**

#### Response after induction 27 (13.85%) 168 (86.15%)

Normal 4 (10.0) 36 (90.0)

Pathologic 21 (15.7) 113 (84.3) 0.598 0.193-1.857 p=0.374

VUS 2 (9.5) 19 (90.5) 1.056 0.177-6.297 p=0.953

Final response 82 (59.85%) 55 (40.15%)

Normal 11 (45.8) 13 (54.2)

Pathologic 62 (64.6) 34 (35.4) 0.464 0.188-1.147 p=0.096

VUS 9 (52.9) 8 (47.1) 0.752 0.216-2.614 p=0.654

**Table S4.** Analysis of treatment response and mutations in the two most mutated genes in the cohort: TP53 and PIK3CA. Specific or shared mutations in these genes with others were compared with the clinical responses: complete response (CR) and partial response (PR)/ stable disease (SD). p-value significant if p<0.05 and size effect indicated by the odd ratio (OR) with 95% confidence interval (CI). 195 (83.3%) and 137 (58.5%) patients were evaluable after induction chemotherapy or randomization respectively.

**Response after induction Final response**

**CR PR/SD CR PR/SD**

**Variable N (%) N (%) N (%) N (%)**

***TP53* mutated**

Presence 15 (12.7) 103 (87.3) 54 (62.8) 32 (37.2)

Absence 12 (15.6) 65 (84.4) 28 (54.9) 23 (45.1)

P, OR (95% CI) p=0.571, OR=1.268 (0.558-2.879) p=0.363, OR=0.721(0.357-1.458)

**Only *TP53* mutations**

Presence 15 (13.8) 94 (86.2) 52 (64.2) 29 (35.8)

Absence 12 (14.0) 74 (86.0) 30 (53.6) 26 (46.4)

P, OR (95% CI) p=0.969, OR=1.016 (0.449-2.303) p=0.213, OR=0.643 (0.321-1.289)

***PIK3CA* mutated**

Presence 4 (21.1) 15 (78.9) 8 (66.7) 4 (33.3)

Absence 23 (13.1) 153 (86.9) 74 (59.2) 51 (40.8)

P, OR (95% CI) p=0.344, OR=0.564 (0.172-1.847) p=0.615, OR=0.725 (0.207-2.538)

**Only *PIK3CA* mutations**

Presence 4 (40.0) 6 (60.0) 6 (85.7) 1 (14.3)

Absence 23 (12.5) 161 (87.5) 76 (58.5) 54 (41.5)

P, OR (95% CI) p=0.024, OR=0.214 (0.056-0.817) p=0.185, OR=0.235 (0.027-2.005)

**Table S5.** Analysis of treatment response and TP53 mutations according to Poeta’s and Neskey’s (EAp53) classifications. Distinct groups of TP53 mutations were compared with clinical responses: complete response (CR) and partial response (PR)/ stable disease (SD). p-value significant if p<0.05 and size effect indicated by the odd ratio (OR) with 95% confidence interval (CI).

**Response after induction Final response**

**CR PR/SD CR PR/SD**

**Variable N (%) N (%) N (%) N (%)**

**Poeta’s classification**

Non-disruptive 5 (12.2) 36 (87.8) 21 (61.8) 13 (38.2)

Disruptive 11 (12.1) 80 (87.9) 40 (61.5) 25 (48.5)

P, OR (95% CI) p=0.986, OR=1.010 (0.327-3.120) p=0.982, OR=1.010 (0.430-2.370)

**EAp53 classification**

Low-risk 4 (22.2) 14 (77.8) 9 (64.3) 5 (35.7)

High-risk 5 (9.4) 48 (90.6) 26 (61.9) 16 (38.1)

P, OR (95% CI) p=0.171, OR=2.743 (0.648-11.615) p=0.873, OR= 1.108 (0.315-3.898)

**Figure S1.** Consort diagram reporting the dropout during the study. From 519 enrolled patients in NCT00716391 clinical trial, 362 diagnostic FFPE blocks were collected and after several quality control steps, 234 were used for the final analysis.

**Figure S2.** Kaplan-Meier survival curves for *TP53* mutated samples. *TP53* mutations were classified according to Neskey et al. in low-risk or high-risk mutations and compared with overall survival (OS) (**a**) and progression free survival (PFS) (**b**). Same analysis was done with Poeta et al. classification in non-disruptive or disruptive mutations for OS (**c**) and PFS (**d**). Median with 95% confidence interval (CI), log rank test p-values and hazard ratios (HR) with 95% CI, are shown in each plot.
